# How can white marble provenance studies change our perception of the stone trade in the Roman Empire: analysing inland Thrace, a *terra incognita*

**DOI:** 10.1057/s41599-025-06228-2

**Published:** 2025-11-26

**Authors:** Vasiliki Anevlavi

**Affiliations:** Austrian Archaeological Institute, Austrian Academy of Sciences, Dominikanerbastei 16, 1010 Vienna, Austria

**Keywords:** Complex networks, History

## Abstract

Provenance studies of ancient white marble provide essential insights into the economic, cultural, and technological dimensions of the Roman Empire. By identifying the geological and geographical origins of marble, such studies illuminate production patterns, trade dynamics, and elite consumption practices across regions. Archaeometric approaches—especially petrography, geochemistry, and isotopic analysis—have become indispensable in this context, particularly when direct quarry evidence is absent. These methods bridge the gap between material science and archaeology, enabling a deeper understanding of how marble circulated, how it was selected, and how it was valued. Despite significant advances in the Western provinces and Asia Minor, the Balkans—and especially Roman Thrace—remain a *terra incognita* in marble provenance research. This study addresses this gap by investigating the production and distribution of white marble in Thracian cities, villas, and sanctuaries, using a combined archaeological and archaeometrical approach. The findings contribute to reconstructing local quarry exploitation, regional exchange systems, and the integration of Thrace into broader imperial markets. Through this case, the study highlights the significance of marble as both an economic resource and a cultural marker, revising current models of the Roman stone trade in the Eastern Mediterranean.

## Introduction

For decades already, scholars have been refining marble provenance studies to investigate marble resources, ancient trade routes, object authentication, and more. These analyses aim to identify the geological and geographical origins of the raw materials used in stone architecture, sculpture, and inscriptions, and other archaeological objects (Anevlavi and Prochaska, 2021). By knowing the main source of marble and the provenance of an object or a monument, several aspects of the research on these artefacts can be elucidated. By investigating the provenance of the material, it aims to uncover insights into the interconnections between cities regarding external relations, such as religious and economic monuments, as well as structures associated with servitude. The study also explores how the use of luxurious marble reflected elite representation and social status. Furthermore, the study considers the economic implications of marble quarrying, recognising it as both a key driver of technological innovation and a major component of the ancient economy. This paper presents an innovative and transdisciplinary approach to analysing the production and use of white marble in Roman Thrace. It uses a combination of archaeological and archaeometrical methods, offering a comprehensive and multidisciplinary investigation into marble’s utilisation and provenance determination in the Roman Balkans and Thrace. This research laid the groundwork by providing a fundamental understanding of marble, its archaeological significance, and the relevant literature review. The study of Roman Thrace, applying a comprehensive archaeometric and archaeological approach, offers the first systematic investigation of the region’s marble economy and provides a novel framework for understanding the balance between local resource use and imported material within broader Roman trade dynamics. The subsequent exploration focused on establishing archaeological and archaeometric frameworks, investigating the characteristics of quarries and extraction processes, providing historical overviews of the Roman Balkans and Thrace, and meticulously studying geological aspects in present-day Bulgaria.

### Terrae incognitae

Provenance studies of white marble aim to combine the ancient disciplines of archaeology and economic history with geology and the applied analytical techniques of petrography and geochemistry. In recent years, our research has focused on combining geological samples from local quarries and outcrops and evaluating all archaeometric data of the marble sources under consideration. Research has focused on developing techniques and methodologies for material characterisation and provenance determination over the last decades, when scientific marble provenance was established in archaeology. Advances in archaeometric (e.g. geochemical) methods and petrography have allowed marble artefacts to be provenanced with a high degree of confidence (Taelman, 2022). This contributes to helping answer questions about the regional and long-distance trade of white marble, which was an essential component of the economic history of the Roman Empire. Typical Roman developments and urban settlement structures, such as houses and villas, began to appear. In addition, urbanisation, in combination with the monumentalisation of architecture, created a large-scale market demand for building materials, specifically targeting durable stones such as white marble. Therefore, the high demand for marble prompted further expansion of existing quarries and the establishment of new stone quarries (Long, 2017). Archaeology has usually studied the Roman marble economy on a case-by-case basis, i.e. based on a single monument, assemblage or site. There is undeniable value in this case-study approach; certain questions can only be answered by examining the data on the provenance of the marble in its entirety (Taelman, 2022). Russell (2013a and 2013b) presented the first overview of the economics of the Roman Stone Trade, introducing evidence from across the Roman Empire. This study targets the Eastern Mediterranean region, with a particular focus on inland Thrace since no previous provenance studies have been conducted in the region. It aims to provide an overview of the marble trade, starting with case studies (e.g. Philippopolis, Montana, Strymon Valley, etc.) and establishing evidence for the general stone trade overview of the province.

Researchers have focused on various parts of the Empire; however, the Thracian quarries have received much less attention and were previously not wholly investigated. Through investigation of the Thracian quarries and Roman artefacts in the main urban centres in the region of Thrace, theories regarding marble trade and the cultural and technological transfer between the Roman world and Thrace are examined. Within this research, the ancient white marble quarries of present-day Bulgaria are explored, with particular attention to the demand for marble in urban architectural settings and the choices and quality of materials used in buildings and other objects, such as sculptures. Possible different choices of marble in comparison with the building types are underlined.

### The example of Roman Thrace

**Fig. 1:**
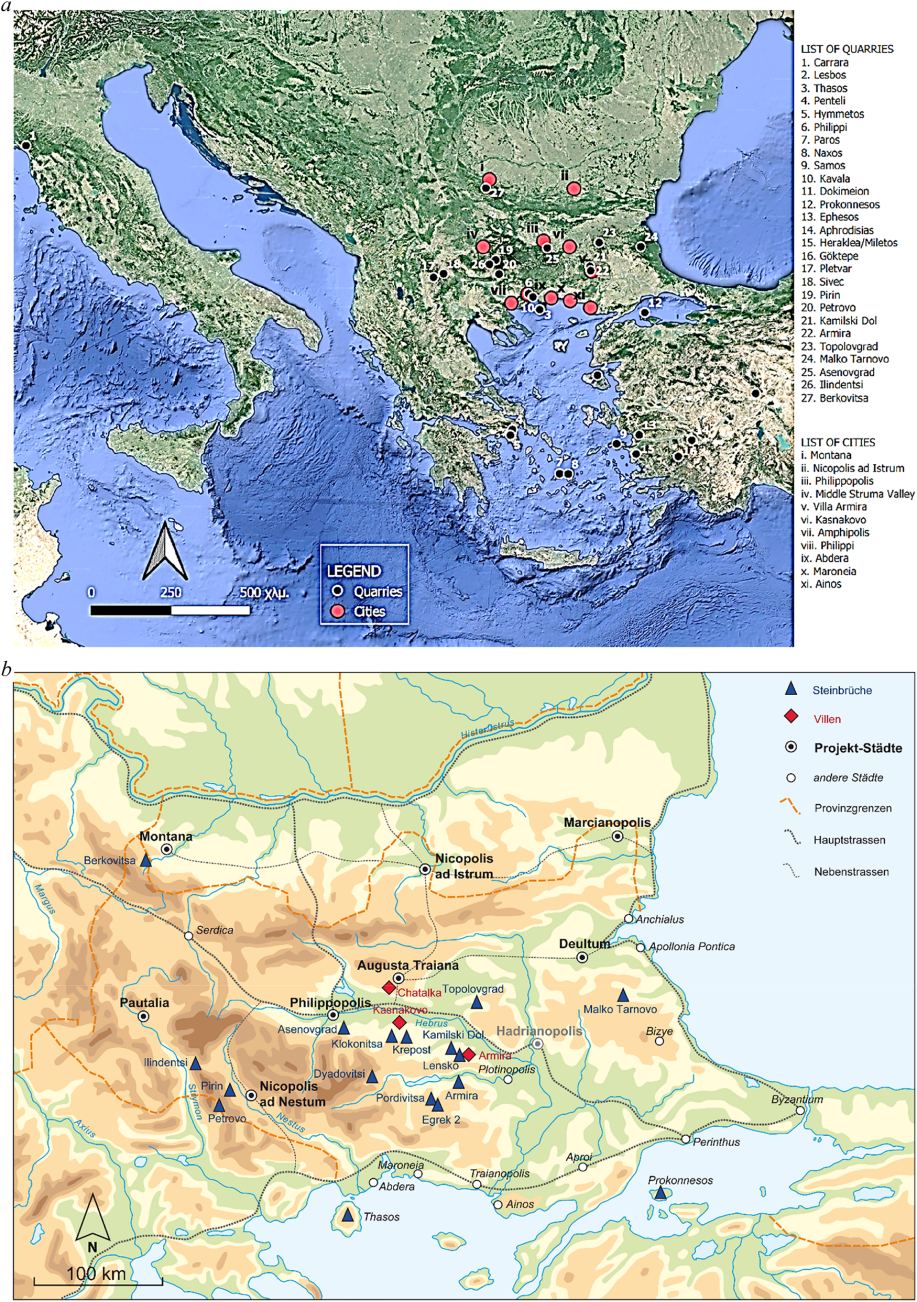
**a** Μap of the most important quarries in the Eastern Mediterranean and cities of Roman Thrace (© T. Georgotas and V. Anevlavi; ed. by the author) **b** Map of Roman Thrace indicating the locations of investigated quarries (blue triangles), villas (red diamonds), and key project cities (black squares). Also shown are other urban centres, main and secondary roads, and provincial boundaries. The map visualises the spatial distribution of marble sources and archaeological sites relevant to the study, with particular emphasis on inland connectivity. Legend translation: Steinbrüche → Quarries; Villen → Villas; Projekt-Städte → Project cities; andere Städte → Other cities; Provinzgrenzen → Provincial borders; Hauptstrassen → Main roads; Nebenstrassen → Secondary roads (© ÖAI/ÖAW).

Specifically, during the first century AD, the Danubian Limes started coalescing when the Roman Empire took Thracian lands south of the River Danube and the provinces of Thrace, Lower and Upper Moesia, becoming an important part of the eastern Roman Empire (Fig. 1a) A new Roman province, Moesia, was established on the Balkan Peninsula in 15 AD. It was the incorporation of all the *appoikiai* (the ancient Greek cities) on the Western Black Sea coast, north of the Balkan Range and their hinterlands. Later, the province of Moesia was divided by Domitian in 86 AD. The Eastern part, including all the ports, was separated and named Moesia Inferior. Until the fourth century AD, the area closer to Constantinople grew in importance, and port towns were established on the Black Sea. Trade routes were developed from these towns through Serdica (Sofia) to central Europe and the Adriatic. The Danube was the northern frontier of the Eastern Empire for centuries and was the base for military garrisons and campaigns into Dacia (southern Romania) (Boteva, 2014).

Within the territory of the province of Thrace (Fig. 1b) and its surrounding regions, some of the most prominent, renowned and investigated marble sources of antiquity are located (e.g. from the islands of Prokonnesos or Thasos). These marbles were widely exported throughout the empire, but we can also trace the marble from these islands to larger inland cities of Thrace (Minchev, 2012). In contrast to these sources, the numerous marble deposits in the interior, particularly in present-day Bulgaria, have received far less attention. Evidence of Roman marble production in the inland territory of Thrace is known in different areas of the Rhodope Mountains, in the Strandza Mountains and the northwest Balkans near the modern town of Berkovitsa (Bulgaria) (Petrova and Ivanov, 2008). Initial analyses indicate that these marbles were not only employed in the province itself but were also exported. A notable example is Berkovitsa marble, which has been identified at Felix Romuliana (present-day Serbia), where it was used for floor slabs and sculptures such as the ‘Boar-hunt’ relief (Prochaska and Živić, 2018). Further analyses planned in the lower Strymon Valley (present-day Serres, Greece; 2026–2027) will explore the potential exportation of local marble into the province of Roman Macedonia. On the other hand, architecture and sculpture in the province of Thrace reveal striking connections to Asia Minor (Dimitrov, 2018).

The timeframe chosen for this study is from the first to the third century AD. During this period, crucial changes concerning the architecture, statuary, etc., have been recorded in inland Thrace. The previous centuries have been excluded due to the occasional use of marble (Ognenova–Marinova, 1984). In earlier periods (from the fourth to the first century BC), the use of marble in the region was limited and highly selective, mainly employed for sculptures, architectural details, and inscriptions (Ognenova–Marinova, 1984), with notable examples in grave architecture (Stoyanova, 2015). In most cases, however, local stones such as granite, limestone, and sandstone were preferred for construction purposes (Stoyanova, 2002). By contrast, Late Antiquity presents a markedly different scenario, characterised by the widespread reuse of earlier materials (spolia) and the predominant use of Prokonnesian marble in ecclesiastical architecture, indicating a shift in both material supply and ideological priorities (Vanderheyde and Prochaska, 2011). It should be mentioned that the Thracian coastal sites are intentionally excluded from this project, as due to their Greek tradition, they reveal pretty different usage patterns of marble in chronological and functional respects.

A set of broader questions concerning the role of marble provenance in archaeological interpretation drives this study. Why is it essential to determine the geological origin of ancient marble? What can provenance studies reveal about economic systems, social hierarchies, and cultural interactions across the Roman Empire? More specifically, how can we address the existing geographical and methodological gaps in marble research by focusing on underexplored regions? Roman Thrace, a largely neglected area in provenance scholarship, offers an ideal testing ground for such questions. Its rich archaeological material, diverse local marble sources, and complex trade dynamics make it a powerful case study for rethinking the balance between local resource use and imperial connectivity. As such, the region not only illuminates its own economic and cultural history but also offers a valuable example (Erdkamp and Verboven, 2015) for future marble provenance research by highlighting the dynamic interplay between local resource exploitation and the selective importation of external materials. Rather than serving as a fixed model, Roman Thrace offers a comparative lens for examining similar mechanisms in other provinces where complex economic entanglements shaped the use and movement of stone materials. Beyond its regional scope, this study contributes to broader debates on material economy in the Roman world (Perkins, 2000) by challenging contradictions between local and imperial supply systems and emphasising the active role of provincial centres in shaping trade networks. Furthermore, the integrated archaeometric methodology presented here (combining petrography, isotopic analysis, and multivariate statistics) can be adapted for comparative application in other regions, allowing for better reconstructions of economic and cultural connectivity in the Roman Empire.

## Methodology

The heterogeneity of the analytical data and the large number of quarries and sources of marble production in antiquity require a combination of different methods for a reliable analysis of the provenance. Microscopic analysis remains fundamental to this study, given the core aims of identifying both primary and secondary mineralogical components of the marble, assessing rock textures, estimating crystal dimensions, and detecting accessory inclusions (Blackburn and Dennen, 1994). Petrographic assessments were carried out on thin sections (30 µm thick) using a Kern OPO 185 polarising microscope. These sections were compared against the comprehensive thin section archive curated by Prof. Prochaska at the Austrian Archaeological Institute (ÖAI/ÖAW), encompassing roughly 350 samples from both ancient and modern quarrying locations.

The integration of stable isotope and geochemical techniques forms a critical part of the provenance protocol. Special emphasis is placed on analysing the oxygen and carbon isotope ratios (δ^13^C and δ^18^O), as established in provenance research by Craig and Craig in 1972, and further developed in subsequent decades (Polikreti, 2007; Attanasio et al., 2006). These isotopic signatures reflect sedimentary and metamorphic histories, making them diagnostic of specific marble types. The methodological advantage lies in the minimal sample quantity needed (approx. 0.2 mg), facilitating analysis even of smaller archaeological artefacts. In this project, the isotopic characterisation was performed at the Bayerische Staatssammlung für Paläontologie in Munich, using CO_2_ extraction via phosphoric acid reaction at 72 °C, interfaced with a Finnigan Gasbench II and measured on a Finnigan Delta-plus mass spectrometer operating in continuous He flow mode. Calibration was performed using in-house standards.

To further enhance discrimination between marble sources, trace element analysis via inductively coupled plasma mass spectrometry (ICP-MS) was also employed. This technique provides quantification of elements at low and sub-ppm concentrations, including Mg, Mn, Sr, Fe, La, Ce, Cd, Ba, Y, Yb, and U, which have demonstrated strong provenance potential. Approximately 0.1 g of each powdered sample was digested in hot concentrated nitric acid (HNO_3_) for around five minutes. This selective dissolution targets carbonate and soluble mineral phases while leaving silicate contaminants largely unaffected. The analysis was performed on an Agilent 8800 ICP-QQQ mass spectrometer, calibrated with Merck VI standards, with JLs-1 limestone used as the internal reference. The laboratory procedures were carried out at the Department of Chemistry, TU Wien. This analytical scheme, when applied alongside traditional parameters and supported by multivariate statistics, has demonstrated high efficacy in distinguishing marble origins (Prochaska and Attanasio, 2021).

Given the high dimensionality of the geochemical and isotopic datasets, comprehensive statistical processing was essential. Multivariate discriminant analysis was employed to assess the combined dataset, using the software packages STATISTICA and SPSS. These programmes enable both quantitative assessment and graphical visualisation of group memberships (Anevlavi and Prochaska, 2021; Anevlavi et al., 2022). Discriminant functions were calculated to allocate samples into quarry-specific groups based on their chemical profiles, assigning probabilities to each classification. STATISTICA allowed the derivation of a reduced number of discriminant functions from the broader variable set, enabling visual and numerical interpretation of sample groupings using factor plots. Consistent variable selection across datasets ensures methodological robustness.

The newly collected dataset regarding Roman Thrace includes approximately 300 geological and more than 400 evaluated archaeological samples, analysed petrographically, geochemically, and statistically in the laboratory of the Austrian Archaeological Institute of the Austrian Academy of Sciences. Although over 400 samples were evaluated in the course of this study, the results presented here focus on a representative selection drawn from many sites that exemplify key patterns in marble use and provenance attribution across Roman Thrace. In total, approximately 900 artefacts were sampled during the course of the project, and their evaluation is currently ongoing as part of continued research. The analytical results were systematically cross-referenced against the extensive marble reference database held at the Austrian Archaeological Institute. A large number of the principal white marble sources in Bulgaria have been systematically sampled, including Berkovitsa, Asenovgrad, multiple locations in the southeastern Rhodope region (such as Armira and Kamilski Dol), as well as Petrovo, Pirin, and other relevant outcrops (Anevlavi et al., 2022). This curated and continuously expanding database allows confident sourcing of archaeological materials and supports the broader interpretive framework of this study.

## Selected case studies and results

The paper of Andreeva et al. (2024) introduces preliminary results, highlighting outcomes of a comprehensive marble provenance study in Inland Thrace, a region previously understudied archaeologically in terms of marble usage. The project targets marble artefacts from cities, villas, and sanctuaries dated to the 1st–3rd centuries AD. Systematic sampling and analysis show that many architectural and sculptural elements were made from local white marble, especially from the Rhodope Mountains and Kamilski Dol, with some items also crafted from imported marbles such as those from Attica, Thasos, Prokonnesos, and Pletvar. Case studies from sites like Villa Armira and Kasnakovo demonstrate both local quarry use and selective importation of prestigious marble for elite expression. Cities like Philippopolis (Plovdiv), Augusta Traiana (Stara Zagora), and sites in the Strymon Valley (Struma Valley) reveal a mix of local and imported sources, suggesting a complex network of quarry exploitation and trade. The study emphasises the region’s integration into wider Roman marble distribution systems.

**Fig. 2:**
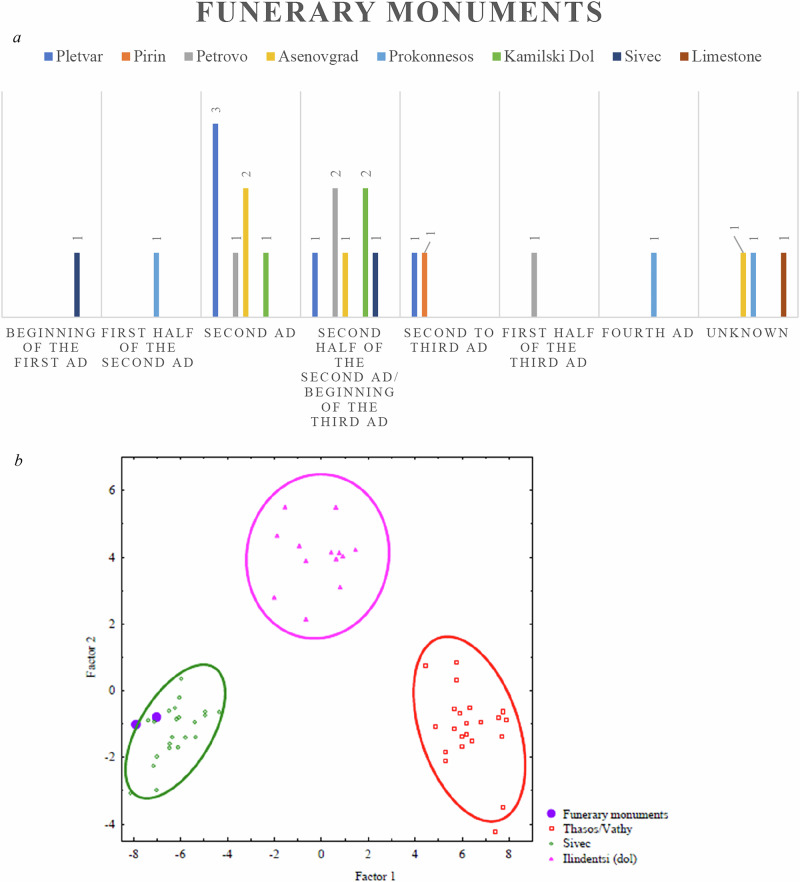
**a** Chronological distribution of the provenance of funerary marble artefacts from the Strymon Valley (present-day the region of Blagoevgrad/Bulgaria). Each bar represents a distinct marble type identified for a given period, allowing for direct comparison of material choices across time. The grouped format highlights the diversity of local and imported stones in the funerary landscape of the region (ed. by the author) **b** Biplot of Factor 1 and Factor 2 from the multivariate discriminant analysis, illustrating the geochemical and isotopic characterisation of selected marble samples. The plot includes dolomitic samples of Thasos/Vathy and Ilindentsi and Sivec marble, with funerary monuments from inland Thrace shown for comparison. The following elements and isotopic values were used in the statistical evaluation (STATISTICA and SPSS): Mn, Mg, Cr, Cd, V, Fe, Sr, La, Ce, Yb, U, δ^18^O, and δ^13^C (ed. by the author).

A study investigates the provenance of white marble used in Roman funerary monuments from the Struma Valley, a key crossroads between Roman Macedonia and Thrace. Most artefacts examined were made from locally or regionally sourced white marble, particularly from the Pletvar, Asenovgrad, and Petrovo quarries. Pletvar marble was the most commonly used, especially during the 2nd century AD (Fig. 2a). Other notable sources included Kamilski Dol and Pirin, while a smaller number of monuments were made from imported Prokonnesian marble. Two examples were also identified as Sivec dolomitic marble (Fig. 2b). The results highlight a strong reliance on local and regional marble sources, suggesting a well-integrated network of quarrying and trade across provincial boundaries. The presence of shared stylistic features and recurring iconographic themes indicates the activity of regional workshops, possibly supported by mobile craftsmen. These findings reflect the economic and cultural significance of marble production and distribution in this part of the Roman Empire (Anevlavi et al., 2025b).

**Fig. 3:**
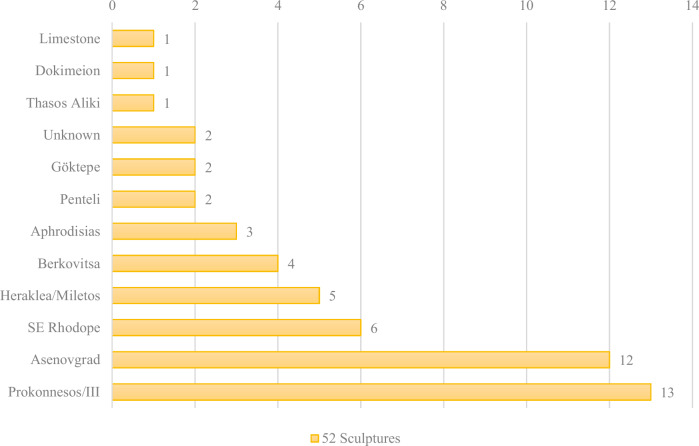
Distribution of identified marble sources across sampled objects (after Anevlavi et al., 2025; ed. by the author).

Regarding the sculpture’s stone trade, the white marble provenance study from Philippopolis (modern Plovdiv) revealed a balanced reliance on both local and imported marble sources, highlighting the city’s integration into regional and supra-regional trade networks. Approximately 42% of the analysed sculptures were made of local marble, primarily from the Asenovgrad and SE Rhodope regions, demonstrating the suitability and availability of local raw materials for fine sculptural work. Imported marbles, constituting 52% of the samples, originated from prominent sources such as Prokonnesos, Aphrodisias, Heraklea/Miletos, Penteli, Göktepe, Dokimeion, and Thasos (Fig. 3). This diversity illustrates Philippopolis’s extensive economic and cultural connections within the Eastern Mediterranean. Notably, Prokonnesian marble was dominant among the imports, used for both portraits and divine statues. The coexistence of local and imported materials across all sculpture categories suggests that material choice was influenced by availability, cost, and symbolic value, reflecting both local craftsmanship and elite participation in broader imperial markets (Anevlavi et al., 2025). Other publications that have been taken into consideration are: Anevlavi et al., 2019; Anevlavi et al., 2022; Anevlavi et al., 2025a; Anevlavi et al., 2025b; Katsarova et al., 2024; Andreeva et al., 2025; Anevlavi et al., accepted.

## Discussion

The following discussion draws directly on the analytical results presented in Section ‘Selected case studies and results’, synthesising the data from each case study to explore broader patterns in marble use, trade dynamics, and cultural exchange in Roman Thrace. By contextualising the identified marble types, object functions, and distribution patterns, the aim is to interpret how these material choices reflect local resource strategies, socio-economic structures, and the province’s integration into wider imperial networks.

Trade is an exchange developing system related to the production and consumption of products transported over small or longer distances (economic point) (Rodrigue, 2020). Defining the trade from the point of view of classical antiquity, it is an external exchange of products related to barter, sale or gift exchange. The forms of trade can be differentiated according to the organisation, exchange arrangements, redistribution, society development, etc., under the umbrella of cultural interactions and connectivity (von Reden, 2014, p. 7363). It could be related to money exchange, peaceful selling and buying of products, gift exchange, piracy and forms of violent acquisition (von Reden, 2014, p. 7363). In terms of the stone trade, primarily, Ward–Perkins introduced the different trade models in marble studies (one of the first studies is related to Tripolitania and its marble trade, as of Ward–Perkins, 1951), while Russell, in 2013, created a milestone in this category by collecting and unifying different aspects of the Roman stone trade from across the Roman Empire (Russell, 2013a). Most recently, Taelman, via a quantitative and diachronic study, presented the latest data and theories of the field (Taelman, 2022).

### Regional self-sufficiency and material choices in Roman Thrace

Examination of various marble items occurring in the province of Thrace (inland) enables us to make several remarks concerning the used material and the product destination (Table 1). The integration of geological samples from local quarries and outcrops with the comprehensive evaluation of archaeometric data provides critical insights into regional and supra-regional white marble trade, a key facet of the Roman Imperial economy and its historical development. In this study, local quarries are defined as those located within approximately 20–30 km from the city, following Russell’s (2013a) model of urban access to nearby resources. Sub-regional quarries lie within a 40–50 km radius, while regional quarries extend up to 260–270 km from the city. Quarries beyond this range—typically exceeding 270 km—are classified as supra-regional, often including prominent sources in Asia Minor such as Prokonnesos and Dokimeion. The examined artefacts, such as architectural pieces, inscriptions, sculptures, votive plates, etc., complete the use of marble and the trade policy. The size of the objects is a crucial aspect. It can be observed that local material is used for the large construction blocks, while for smaller objects (sculptures, inscriptions, etc.), a different approach can be seen using a large variety of supra-regional stone sources. Furthermore, the votive plates were either part of a local production or partially made of imported marble. The votive reliefs could be a production made of waste material. The large (or larger) waste piece resulting from preparing a big boulder for either architecture or sculpture could have been used to make these reliefs.

**Table 1:** Summary of marble types attested at selected sites in Roman Thrace and their associated object categories.

Site	Marble type(s)	Object type(s)
Philippopolis	Asenovgrad, Mostovo, Prokonnesos, Pavonazzetto	Architectural elements, theatre, agora, stadium, sculptures
Montana	Berkovitsa	Inscriptions, sculptures, and architectural elements
Serdica	Berkovitsa	Frieze, architectural blocks
Nicopolis ad Istrum	Berkovitsa, Asenovgrad, local limestone	Votive plates, inscriptions
Villa Armira	SE Rhodope (Kamilski Dol, Armira)	All architectural elements
Kasnakovo	Penteli, Thasos, Asenovgrad, local limestone	Sculptures, daily objects
Pautalia	Various white marbles, local limestone, Pavonazzetto	Architectural elements, sculptures, votive plates, and inscriptions
Augusta Traiana	Local marble, Prokonnesos, local limestone	Architectural elements, sculptures, and inscriptions
Strymon Valley	Pletvar, Petrovo, Pirin, Asenovgrad, Prokonnesos	Funerary monuments
Felix Romuliana	Berkovitsa	Sculptures, floor slabs
Alexandroupolis	Asenovgrad (?)	Votive plate

The results presented in this table and throughout the paper reflect the current state of analysis as of March 2025. Further archaeometric investigations and case studies are ongoing and will be incorporated in future publications.

**Fig. 4:**
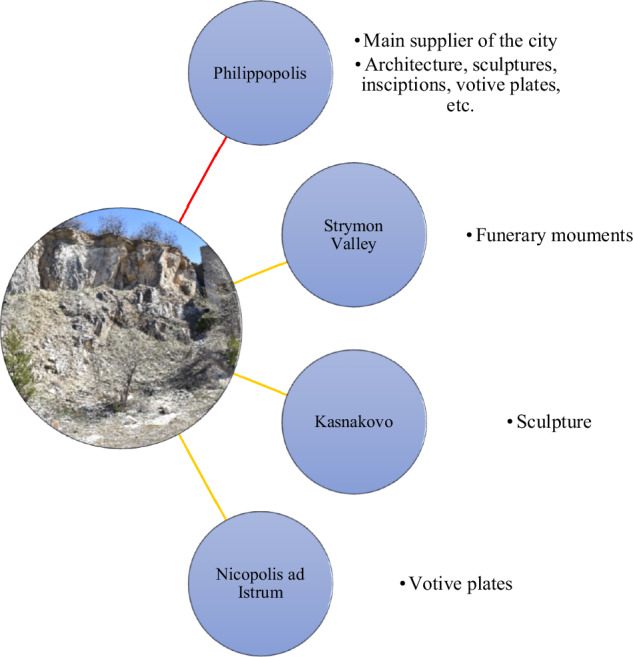
A graphic presentation of the Asenovgrad marble and its distribution within the province of Thrace. The differing line colours depict the quantity of objects made in Asenovgrad to the cities (yellow: 1–10 objects; blue: 11–49 objects; red: more than 50 objects) (ed. by the author).

The case study of Thrace, including multiple focused case studies (e.g. Anevlavi et al., 2025; Prochaska et al., 2025; Andreeva et al., 2024; Anevlavi et al., 2022) projects the phenomenon of ‘localism’ by prioritising the local raw material and the local production and consumption of goods (Fig. 4). However, on certain occasions, the province kept its connectivity with large marble producers of the Eastern Mediterranean, such as Prokonnesos, Aphrodisias, Penteli, etc., for specific artefacts, e.g. mainly elements of decorative architecture and sculptures (Anevlavi et al., 2025).

In order to set the evidence in context, the analysis results from a series of sites are introduced, showing solid results on extensively using local sources with the occasional use of imported material. In the northwest part of the province, Berkovitsa marble produces an extensive range of objects, including altars, statue bases, inscriptions, sculptures, votive reliefs, and funerary monuments. This approach shows that this material was available and usable for a large artefact typology, from small votive reliefs to full-size sculptures. Significant architectural elements have been found outside Berkovitsa and Montana (Bulgaria), specifically in Serdica and Philippopolis. The architrave–frieze from Serdica (FWM0094) as well as the coffer from Philippopolis (FWM0547) prove firstly the production of monumental architectural elements and, secondly, their high-quality craftsmanship based on the detailed decorated execution (Andreeva et al., 2024). Finally, these products were exported regionally. As mentioned before, Berkovitsa marble was also used for small objects. The case study of the Sanctuary of Artemis/Diana, the area of Nicopolis ad Istrum, confirmed the presence of this material in the form of votive plates (three in total) (Andreeva et al., 2024). Investigations from Felix Romuliana (present-day Serbia) showed the appearance of the Berkovitsa marble in the form of a sculpture, the ‘Boar-hunt’, and architectural pieces (floor slabs). The modern road distance between Berkovitsa and Felix Romuliana is approximately 150 km (Prochaska and Živić, 2018).

A similar extensive local use can be seen in the southwestern part of the province. The geographical location of the Strymon Valley represents a major crossroad of the ancient network that ensured the connection between the Aegean world and the Balkan hinterland. It crossed the borders between Roman Macedonia and Thrace and is a link between East and West, crossed by the *Via Egnatia* in the southern area. The majority of the funerary monuments are made of Pletvar marble (North Macedonia), while other artefacts can be detected from imported marble. An interesting mix of local sources is demonstrated: sources in direct proximity to the Strymon Valley (Petrovo, Pirin) from the Province of Thrace and Asenovgrad (hinterland of Philippopolis). One of the big antique supra-regional marble sources, Prokonnesos, is also represented in three funerary monument samples. This combination of imported marble material and local production establishes a strong level of economic integration and development by exploiting the local raw material sources in the region (Anevlavi et al., 2025b).

Villa Armira is one of the examples of a complete use of local material from the SE Rhodope (Armira and Kamilski dol quarries). All types of architectural elements, including decorative pieces, are made of these sources (Anevlavi et al., 2022; Anevlavi et al., 2023). The Kasnakovo case study underlines the local use of marble/limestone for architecture and daily objects. Despite the absence of sculptures in Villa Armira, in Kasnakovo, the small analysed group showed imported materials such as Penteli and Thasos, and only one sculpture is made of local marble from the Asenovgrad region (Katsarova et al., 2024).

Finally, the material from the ancient town of Pautalia (Kyustendil, Bulgaria) and its territory, such as architectural elements (pilaster capitals, etc.), but also sculptures, votive plates, and inscriptions, showed that a combination of limestone and marble was used. Different types of marbles can be observed, ranging from fine to coarse-grained, and they come in various colours, from white to grey and beige shades. Pautalia’s sanctuaries of Hisarlak and Kopilovtsi reveal a large number of votive plates. Similarities to the two previous sanctuaries can be seen in the main use of medium-grained and ultra-fine-grained marbles. A different approach to the use of marble can be observed in the case study of the building complex of the thermae, with mainly architectural elements in addition to inscriptions and sculptures sampled. Different types of marble can be seen, including some coloured sources, such as the Pavonazzetto (Dokimeion), used for architectural elements, such as door frames and thresholds (Andreeva et al., 2024).

Similar preliminary observations can be addressed in the case study of Augusta Traiana. In the Forum, two different types of sources can be observed: local marble and, in the case of the Corinthian capitals, the use of Prokonnesian marble. An interesting variant of this site is the extended use of possibly local limestone for many different purposes, such as architectural elements, sculptures, imperial inscriptions, votive plates, etc. (Andreeva et al., 2024) (Table 2).

**Table 2:** Examples of distances between the local quarries and the ancient cities (ed. by the author).

From (quarry)	To (city)	Distance (in a straight line)
Asenovgrad (main supplier)	Philippopolis	≈20 km
Asenovgrad	Strymon Valley	≈145 km
Asenovgrad	Kasnakovo	≈50 km
Mostovo (secondary supplier)	Philippopolis	≈35 km
SE Rhodope	Philippopolis	≈130 km
SE Rhodope	Villa Armira	≈12 km (Kamilski Dol) or 2 km (Armira)
Berkovitsa (main supplier)	Montana	≈25 km
Berkovitsa	Nicopolis ad Istrum	≈200 km
Marciana (main supplier)	Marcianopolis	≈5 km
Samovodene (main supplier)	Nicopolis ad Istrum	≈10 km

### Connectivity and the role of imported marble in provincial networks

While studying the stone sources and their distribution, it is important to understand the topographical position of the raw material source and its final destination. The aforementioned case studies illustrate the coastal distribution of imported marble, particularly in the coastal cities of Asia Minor. However, inland examples from diverse regions demonstrate that stone materials were transported well beyond coastal areas. Notable inland sites such as Baalbek/Heliopolis (Lebanon), Jerash/Gérasa (Jordan), Tadmur/Palmyra (Syria), and Amman/Philadelphia (Jordan) provide evidence of this extended trade network (Dodge, 1990). In light of this, the province of inland Thrace must now be considered within the broader framework of the inland marble trade. The role of the Danube River as a major transport and communication artery is acknowledged, and its logistical and economic significance for regional connectivity and the movement of imported materials is well recognised (Habaj, 2017; Olson and Krug, 2020). However, a more extensive network analysis of the Danubian corridor will be addressed in forthcoming research, following the completion of additional provenance studies in cities such as Nicopolis ad Istrum. Recent investigations have revealed that stone materials, both local and imported, circulated extensively within the interior of the province, underscoring Thrace’s integration into regional and supra-regional trade dynamics. So far, in the central inland area of the province, in Philippopolis, the sampled artefacts show a preponderance of local material; however, imported marble sources can also be detected (Anevlavi et al., 2025). Various buildings such as the agora, the theatre, the stadium, the Eastern gate, etc. According to the data evaluation, the majority of these constructions are made of local Rhodope marble—Asenovgrad, with several marble combinations such as the use of Prokonnesian marble in the Corinthian capitals of the Eastern gate or the use of local white marble and coloured breccia (Asenovgrad and Mostovo) in the theatre. It is important to mention that only specific types of architectural parts appear to be Prokonnesian imports, and only in places such as the agora and the Eastern gate, while the theatre and stadium are exclusively made of local materials (Anevlavi et al., in preparation). Specifically, the Eastern gate is located in the city’s northeast corner, not the eastern wall. Despite this topographic alteration, the name Eastern gate possibly appears already in Antiquity since the building was constructed on the road to Constantinople (east direction, *ανατολική πύλη*) (Topalilov, 2016). Prokonnesos was one of the main suppliers of the Roman Empire, and the marble was traded in Rome and the Italian peninsula in the second century AD for complete constructions (such as the Arch of Trajan in Ancona, Italy), becoming the main supplier across Italy, coexisting with large quantities of Carrara marble (Taelman et al., 2020; Taelman et al., 2021; Taelman, 2025). The extended use of Prokonnesian marble and many other prominent Roman sources coexist with the erection or restoration of buildings (mainly in the second century AD) (Taelman et al., 2020). Usually, by choosing Prokonnesian marble, craftsmen use the Asia Minor style along with the material in the case of the absence of local traditions (Blagg, 1983).

There is an important internal movement of marble, especially the sources of Asenovgrad and the SE Rhodope (mostly in sculptures and other smaller objects), that pinpoints the use of this high-quality local marble of the Rhodope mountains. As mentioned above, public buildings were often partially the responsibility of imperial projects but mostly were constructed via non-imperial activities, meaning the elites’ funds (Russell, 2013a). The use of Asenovgrad marble can be identified so far in various locations and for various object patterns, such as funerary monuments in the Strymon Valley, votive reliefs at the Artemis/Diana Sanctuary at Nicopolis ad Istrum, and in the form of a sculpture in Kasnakovo. It is possible that this marble was exported to the neighbouring provinces as well. Votive plates from the Archaeological Museum of Alexandroupolis (Greece), found on the South coast of Thrace, share the same microscopic characteristics with the Rhodope material, such as the quarry of Asenovgrad. Further archaeometric investigations will examine the possibilities of exporting the local Thracian sources to different sites.

Moreover, extensive research by Andrianou and Lazzarini around Greek Thrace and Macedonia provided new avenues for exploring marble trade networks and discussions around trade and connectivity between provinces. The analytical work on local quarries and a large group of grave monuments highlighted the local, Thasian and Prokonnesian use of marble (Andrianou and Lazzarini, 2013; 2020; Lazzarini, 2017). The research results presented by Andrianou and Lazzarini (2020) emphasise the possibility of the local usage of marble from the northern area of the coast and the different quarries of Rhodope and the connection of these important sites located along the lower reaches of the Maritsa/Hebros River in present-day Greece and Türkiye. Asenovgrad could be a high-potential source for this group of objects.

Despite the main focus of this study being white marble, the use of local limestone should be acknowledged. Local grey with red veins limestone was used in Kasnakovo for the construction of the Nymphaeum (Anevlavi et al., 2019). A significant source of limestone production is possibly linked to Augusta Traiana, which is extensively used for many types of objects (imperial inscriptions, sculptures, etc.). Unfortunately, the exact location of this source or sources has not been identified yet. Important Roman quarries near Nicopolis ad Istrum (Nikyup, Bulgaria) and Marcianopolis (present-day Devnya, Bulgaria) provided the material for each city, with Nicopolis ad Istrum and its buildings being exclusively made of the local limestone. These locations produced not only architectural products but also votive reliefs, inscriptions, funerary monuments, etc. (Prochaska et al., 2025).

The demand for building materials was enormous, with a preference for hard limestones, marble, and materials aimed at various decorative effects (Russell, 2013a). These choices can be seen in Philippopolis’s case study as well. So far, one of the most prominent uses of limestone/breccia is in the theatre of Philippopolis, with this material being used in various forms and patterns. In addition, miscellaneous limestone objects can be noticed in each location, such as in Philippopolis and the limestone head of a woman (FWM0489) (Anevlavi et al., 2025). It is crucial to underline those objects and buildings that were produced in these local materials, applying local traditions and craftsmanship knowledge. The workshops and artisans demonstrated the technical skill and artistic proficiency necessary to work with local materials, achieving results comparable in quality to those produced using imported marble (Heidegger, 2017). It is also possible that different workshops were responsible for the various materials, but with constant collaboration, as the example of the Philippopolis theatre proves (two different materials, marble and limestone).

The number of imports from different locations, local, sub-regional, regional, and supra-regional, proves that the Roman Empire’s market had no trade barriers, including all the possible directions (Gibbins, 1987), assuming that the relevant funds and networking existed. The imported items needed to be absorbed by the local communities and familiarised with the rules accompanying architecture and art (Fischer, 2007). It could be safely presumed that Philippopolis obtained a critical role in the economic network of the province from the second half of the first century AD onwards, given the increasing flow of local productions and imported goods (Andreeva et al., 2025). The small number of supra-regional quarries could indicate a system of gift exchange between groups or individuals (for example, sculptures). Another theory concerning the ways of handling imported material is the redistribution of goods. The gathering of material from a central person, a dealer, and the distribution of the materials to a third party, most commonly the elites. The last category involves the marketing processes, the use of money and the economy of each area (Gibbins, 1987). Despite not knowing the material costs, it is important to consider the possible connections of the customer to the source as well as the continued and large demand for material, especially from the supra-regional quarries (Russell, 2013a).

Knowing the existing stone material (sources) and their use, Philippopolis gives us a clear picture of the trade supplies and connection between the region and the province. However, despite the multiple imports, especially in the case of sculptures, the lack of well-known important supra-regional quarries (white and coloured) is striking (e.g. Paros, Carrara, etc.). An important aspect of the marble inventory of the city of Philippopolis is the absence of Thasian marble (calcite or dolomite). The early trade connections between Thasos and Thrace have been studied by Archibald (2016). A voyager undertaking a distance of about 2000 km would have been considerably harder to organise in 200 BC or even 200 AD, compared to the latest centuries, due to the difficulties of ensuring safe passage across many, perhaps incompatible, frontiers of regimes. Due to such negotiations, which needed to be embedded in local and inter-regional arrangements, complex patterns of trust and support were involved (Archibald, 2016). Thasian marble appears as scattered elements across the Thracian province. The use of Thasian dolomitic marble (Cape Vathy) can be found in Montana and Kasnakovo in the form of sculptures and Augusta Traiana in the form of column shafts, dated in the Byzantine period (ongoing investigations by the author). In Philippopolis, only one Thasian sculpture has been identified from the calcite source of Aliki. Locations with good transportation networks, such as Thasos, Prokonnesos and Carrara, faced vast supply demand (Russell, 2013a). It is possible that due to the extensive demand for this source, priority was given to other provinces and customers, while inland Thrace was capable of covering the need for marble with local material. Thasos was a free city under the influence of Thrace’s governor immediately after the province’s creation, even if it had strong links with the Macedonian elites (Fournier, 2013). Thasian marble was exported to Samothraki and other neighbouring islands, as well as Asia Minor coastal cities, Southern Greece, Rome, etc. (Laskaridis et al., 2013).

A significant question remains whether the workers and artists travelled with their material. It is possible that, in many cases, architects, masons, etc., were travelling along with the blocks or semi-finished objects. However, the lack of written sources cannot confirm this practice. In many cases, one construction’s variation of architectural styles could indicate this activity (Blagg, 1983). According to related studies, it appears that large orders were attended by specialists (Fischer, 2007). In general, a large number of imports indicates a smaller local marble development in each community. Nevertheless, in the case of Roman Thrace, we see a large-scale development of local stone production in combination with being a consumer of imported art (mainly sculptures). The different levels of Roman influence on architecture, sculptures, etc., led to the blending but also adaptation of local practices and local materials with imported goods, practices, and craftsmanship (Dodge, 1990). Regarding raw stone material, workshops and craftsmanship, certain aspects should be considered, such as the stone’s workability, hardness and condition, colour and purpose of use (Russell, 2013a). In some cases, we see that local workshops copy the sub-regional style of stones which are available in the area. This application by the ‘copyists’ creates the base for the long-lasting late antique architectural decoration fashion (Fischer, 2007).

### Trade infrastructure and the function of emporia in material circulation

A final key point in discussing the trade markets and networking is the facilities and functions of the Emporia. The emporia are important establishments during the study of local economies and product distribution by being organised settings by the official of the cities, supporting trade activities in these cities and their vicinities. They had variability related to their function, origin, lifetime, and local wealth (Tiverios, 2008). These trading posts assisted the connections between the commercial centres via the regional and super-regional networks, promoting the prosperity of local trade (Tiverios, 2008; Boyanov, 2014). The earlier form of emporia during Classical and Hellenistic times led to the development of the emporia in Roman times, especially in the Eastern provinces, by continuing the function near the initial ones. The function of the emporium was based on the laws, as well as taxes and fees of the main administrative cities and their officials. Their locations were often close to provincial borders, thus playing a significant role in distributing local and imported goods. Therefore, emporia could mark the contact zone between foreign merchants and the local population in the frame of interprovincial or interregional production networks (Boyanov, 2014). The emporia would have possibly been the link between the quarry/production centre and the final destination of the customer/centre in certain cases, especially in the more isolated areas of various provinces.

The information on the emporia of Thrace, in combination with the complete set of archaeometric analyses of marble products across the province, could eventually provide us with the missing link between these facilities and the stone trade. The names of five emporia are epigraphically attested in the province of Thrace: Cillae in the territory of Philippopolis; Pizos, Discodurаtеrае and Thuida in the territory of Augusta Traina, and Piretensium in the territory of Nicopolis ad Istrum. Parembole, located 4 km away in the southeast direction from the village of Belozen, Plovdiv region, is attested in both epigraphic and written sources as *mutatio Paramvole* (*Itinerarium Burdigalense*, 333–334 AD) but also as *ἐμπορεῖον* (*Passio Sancti Alexandri*) (Boyanov, 2014). An important and well-studied example from the area of Thrace is the Emporium of Augusta Traiana, the settlement of Discoduraterae. It was established during the reign of Emperor Marcus Aurelius. The epigraphic reference to a member of the city’s elite indicates the connections between the city and the Emporium and strongly suggests its significance for the economy of Augusta Traiana. Later on, the administration of the Emporium was transferred to the city of Nicopolis ad Istrum, while from the third century AD onwards, there is no epigraphic evidence found in the area. Evidence of new construction periods has been dated to the end of the third and the beginning of the fourth century (Boyanov, 2014). Another example of an Emporium is the settlement of Pizus. Epigraphic evidence of this establishment gives information about the market function, with settlers of the provincial governor group or independent settlers with promised benefits (Boyanov, 2014). The inscriptions and the coins recovered from the settlement underline the numerous privileges from the governor to the settlers, as well as the strong connections of the establishment with Augusta Traiana (Boteva, 2000). By analysing, in the near future, the artefacts from Augusta Traiana and Nicopolis ad Istrum, it is possible to connect the other imported objects, such as ceramics and their place of origin, with marble sources of the same areas and their possible stone markets.

Despite the limited information on the different emporia of Thrace, it can be concluded that all these locations played an important role in the networking and marketing of goods within the province, as well as assisting the cities in developing larger territories (Boyanov, 2014). No information is related so far to the stone production and distribution to the local emporia of Thrace. A single example from Cillae, an Emporium near Philippopolis, could start a new discussion on this matter. The statue of the Large Herculaneum Woman, which was found in the area, is made of SE Rhodope marble, and the location of the Emporium, in combination with these results, could indicate the commercial route between the sources of the SE Rhodope and the territory of Philippopolis (Andreeva et al., 2025). In general, this limited information could possibly be explained either to the secondary importance of the stone as a product, and the given priority to other goods (such as grains, wine, etc.) or to the fact that the local production was sufficient enough, covering the needs of the region, and only a small number of products made of supra-regional sources were reaching inland areas.

## Conclusions

The study of Roman provinces or Roman expansions is an issue that raises questions about whether it is a matter of cultural adaptation or the Roman influence on the regions. The spread of Roman culture in the conquered territories was carried out through the military administration and the Roman colonies. In addition, the upper social strata of the provinces also played an important role in the process of their adaptation to the Roman characteristics, while the latter took various dimensions, depending on the geographical area and its characteristics (Chatzopoulos, 2015). The size and the long timespan of the Roman Empire had a great influence on the evolution of art, in combination with the search and use of multiple materials (especially stones) and techniques (Mpouras, 1999). The flourishing markets of the Roman Empire thrived from market protection, which cannot be discussed in detail here.

Trade and markets flourished under the Pax Romana, with products like food, pottery, stones, etc., moving freely around the Mediterranean Sea (Temin, 2012). The architecture during Hellenistic times used the natural rocks available in each location, with the Romans later using and redistributing the geological prosperity of the Mediterranean Sea (Blagg, 1983). Despite the possible earlier start of stone’s importation, the main peak of this trend is dated between the first and the third century AD, with evidence of continuation in the fourth and fifth centuries AD (Russell, 2013a). During this period, various changes and processes were related to the different stone and marble sources and the use of the material (Nogales-Basarrate et al., 2015). In Rome, the two primary materials for usage were travertine and tuff. The use of white and coloured marble soon lost its meaning as a simple material, and eventually, their appearance became a symbol of luxury among the elites (among other stone materials, such as granites, alabasters, tuff and travertine, etc.) (Mpouras, 1999). The demand for distinctive materials found its main support in maritime supply routes, creating connections between major white and polychrome marble quarries in the Aegean, northern Italy, and select areas of Africa. This network served a diverse and geographically extensive trade (Russell, 2018, p. 132).

Hierarchical and social status and prestige were among the main interests of the wealthy members of Roman society. This was achieved via monumental architecture and sculptures, showing their lavish choices for the benefaction of the elites (Taelman et al., 2020). In many provinces, the urban elites, being part of the Roman Empire, constructed and decorated public facilities by donating private funds (Dodge, 1990). However, in the case of building construction, the high costs of the materials most likely were spread among numerous individuals (Russell, 2013a) (For analytical price information in the Roman Empire, see Tonisch (2022) *Omnia Romae cum pretio. Löhne, Preise und Werte im Römischen Reich)*. The Diocletian Edict shows the prices of various stone materials; unfortunately, we have no information on the prices of stones in Thrace. Despite the long distances, various stone materials were exported across the Empire, with the main sources being Iasos marble, Pavonazetto, Giallo Antico, Rosso Antico, Cipollino, Penteli, Paros, Prokonnesos, Aphrodisias, Carrara, etc. (Mpouras, 1999). Notably, these areas comprise high-quality white marble sources that are reasonably accessible (islands or coastal sources), in addition to uniquely coloured materials, or especially strong ones (e.g. granites), which lead to the transportation and trade of possibly carved, finished stone objects. The availability, primarily of raw materials but also of trained craftsmen who were experienced with the particular sources, led to the blooming of the stone trade (Dodge, 1990).

New quarries were opened to cover the demand of urbanisation and monumentalisation of the provinces, or the expansion of the already existing ones was necessary. Although much attention has been directed at those quarries where we have evidence of imperial involvement, most quarries were owned by administrators/owners, who were the municipalities or private individuals (Russell, 2013a). The study of the quarries in the area of Bulgaria provides new information about the importance of the local sources (Asenovgrad, SE Rhodope, Berkovitsa, Petrovo, etc.) and the role played in the local stone production and economy. While these sources have been studied partially in the past, the amount and the high quality of the local quarries of the area add new perspectives to the economic aspects of the regions and the surrounding provinces (Andreeva et al., 2024). It is worth pointing out that most quarries were located near cities, mirroring the pattern of the urbanisation processes—only those quarries with very high-quality materials or exploited by the emperors were more remote (Russell, 2013a). In Roman Thrace, many sources were located near the urban centres, such as Berkovitsa near the city of Montana or Asenovgrad near the city of Philippopolis, providing the immediate necessary number of raw stone materials. Moreover, small-scale localised quarries were also present across the Empire, (a case study of the Ab-u Hayat quarry near Ephesus proves to be a location of local sarcophagi production, as of Anevlavi et al., 2021), and in the case of Roman Thrace, the quarry pits in the Eastern Rhodope (Kamilski Dol and Armira) and the Western part of the province (Petrovo and Pirin) were used for various types of artefacts on a smaller or larger scale (from funerary monuments to villa architectural construction). Not much information is linked to the administration and ownership of these sources (Cenati, in press).

Nogales-Basarrate et al. (2015) use the term ‘visual language’ to describe urban culture and its wide-ranging application in terms of architectural approaches and decorative outlines. Common trends and differences in design and style can be specified by examining the artistic items. In addition, the geographical diffusion of the different styles of objects plays a significant role (Fischer, 2007). A primary factor of material choice is the availability of raw sources either locally or in the vicinity, especially when it comes to building materials (Blagg, 1983). By studying the quarries and their products, we understand the importance of the primary production phase and material choice. It is believed that the main production was done in the quarries by carving objects of standard dimensions and forms, and used as stock material (Ward–Perkins, 1980). As soon as the marble trade was widespread across the Empire, new opportunities were established to create workshops across the Mediterranean (Dodge, 1990). Asia Minor started a new style in the decoration of architecture during the Flavian period, with a rich veneer style, as well as columnar façades, employing local marble—the so-called ‘Marmorstil’, using the German term first applied to it (Fischer, 2007). During the first and second centuries AD, the Romans performed a well-organised marble quarrying and trading system, creating a framework for spreading the material and the style (Fischer, 2007). Each region’s trade and economic development varied due to the geographical expansion and dissimilarities of the Empire (Russell, 2013a). The province of Thrace was mainly independent of marble imports since it is mostly ‘self-sufficient’ by using local sources for various object types. The good marble quality of the local raw stone material, and especially the white marble, appears to dominate every aspect of the stone usage. The sampled artefacts made of imported marble show specific patterns and choices, such as parts for decorative architecture, sculptures, and partly inscriptions and funerary monuments.

In most cases, architectural decoration, statuary, sarcophagi and other objects were used for the luxury business (Fischer, 2007). Depending on the evidence in the Roman cities in Bulgaria, information is given about the character of the buildings, and more specifically, the civic centres, theatres, temples, sculptures and decorative architecture connected with the imperial cult and various gods. By studying the urban context of Roman Thrace, the epigraphic evidence shows the significant role assigned to wealthy members of the city communities in the process of monumentalisation through the honorary inscriptions set up by them as benefactors. Although the extent of the importance of civic euergetism appears to be still quite debatable in the context of the munificence policy of provincial cities, these inscriptions are primary sources for the social, financial and even cultural relationships in the urban context (Kokkinia, 2012).

Overall, the results of this study are very promising and proven successful—they will, without a doubt, revise the current commercial-historical and archaeological models regarding trade and exploitation of white marble in Antiquity, particularly in the Roman imperial period. Once the trans-provincial trade during the Imperial period was established, gaining additional understanding about the scope of the marble trade could be achieved by conducting international studies in the contemporary neighbouring countries. This approach would contribute to a better grasp of the distribution networks of quarries and the overall marble economy.

This study contributes to a better understanding of the economy and the societal structure of the area and may serve as a model for other larger-scale research of local quarries and ancient sites, as well as the connections and the long-distance trade and sub-regional/regional marble markets. The stated overriding objective of the hypothesis-oriented and unbiased research approach is to develop a standard method for the identification of the origin of white marble.

## Data Availability

All published analytical data for the discussed samples are openly accessible via the oeai.METRIX database: https://oeaimetrix.oeaw.ac.at/ (Anevlavi et al., 2025c). This resource comprises data from over 5700 samples from ancient and modern quarries and approximately 4500 artefact samples. The comparative dataset spans major marble-producing regions of antiquity, including Asia Minor, Greece, and the Balkans. It represents decades of collection and analysis under the leadership of Prof. Prochaska and remains accessible via the online platform: https://oeaimetrix.oeaw.ac.at/ (Prochaska, 2021; Prochaska and Attanasio, 2021, 2022; Prochaska et al., 2024).
